# Supplementary material for: Identification of a family of species-selective complex I inhibitors as potential anthelmintics
Source: Nat Commun. 2024 May 8;15:3367. doi: 10.1038/s41467-024-47331-3 (PMC11079024; doi:10.1038/s41467-024-47331-3)
Supplement: Supplementary file 1 — Supplementary Information [file 41467_2024_47331_MOESM1_ESM.pdf]

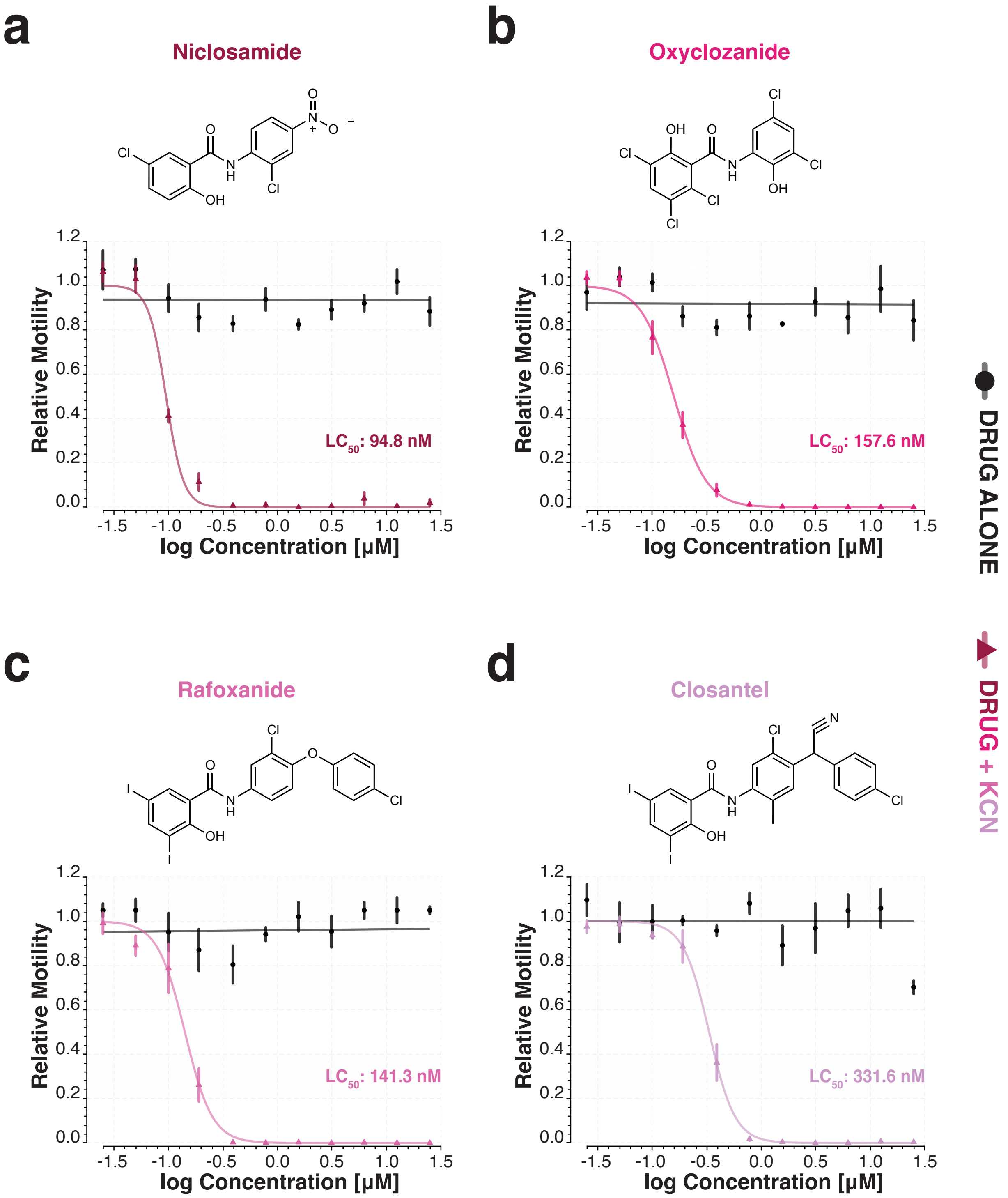

Supplementary Figure 1. Salicylanilide anthelmintics are strongly potentiated by potassium cyanide (KCN). Chemical structures and dose responses for niclosamide (**a**), oxyclozanide (**b**), rafoxanide (**c**), and closantel (**d**) alone and in combination with 200 μM KCN against L1 wild-type *C. elegans* in 15-hour KCN survival assay. LC50 values estimated from dose-response curves are shown for each combination of compounds. Data are the mean of at least three biological replicates; error bars represent SEM.

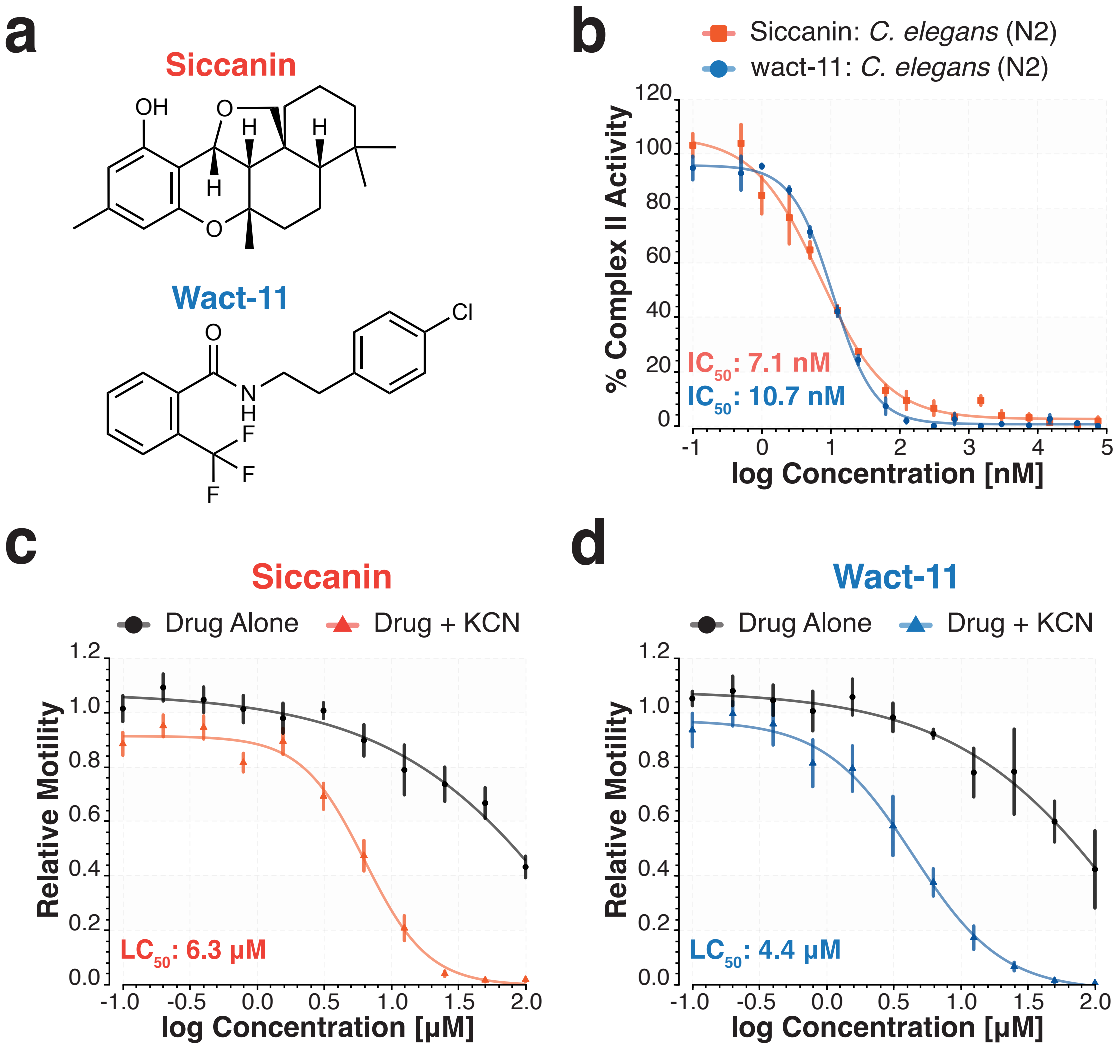

Supplementary Figure 2. The antifungal siccanin inhibits *C. elegans* succinate dehydrogenase (complex II). a. Chemical structures of siccanin and wact-11, two previously reported species-selective complex II inhibitors. **b.** Dose responses of siccanin and wact-11 against the *in vitro* activity of complex II from wild-type *C. elegans* (N2) mitochondria. IC50 values estimated from fitted curves are displayed for each compound. **c**. and **d**. Dose responses for siccanin and wact-11 alone and in combination with 200 μM KCN against L1 wild-type *C. elegans* in KCN survival assay. LC50 values estimated from dose-response curves are shown for each combination of compounds. Data are the mean of at least three biological replicates; error bars represent SEM.

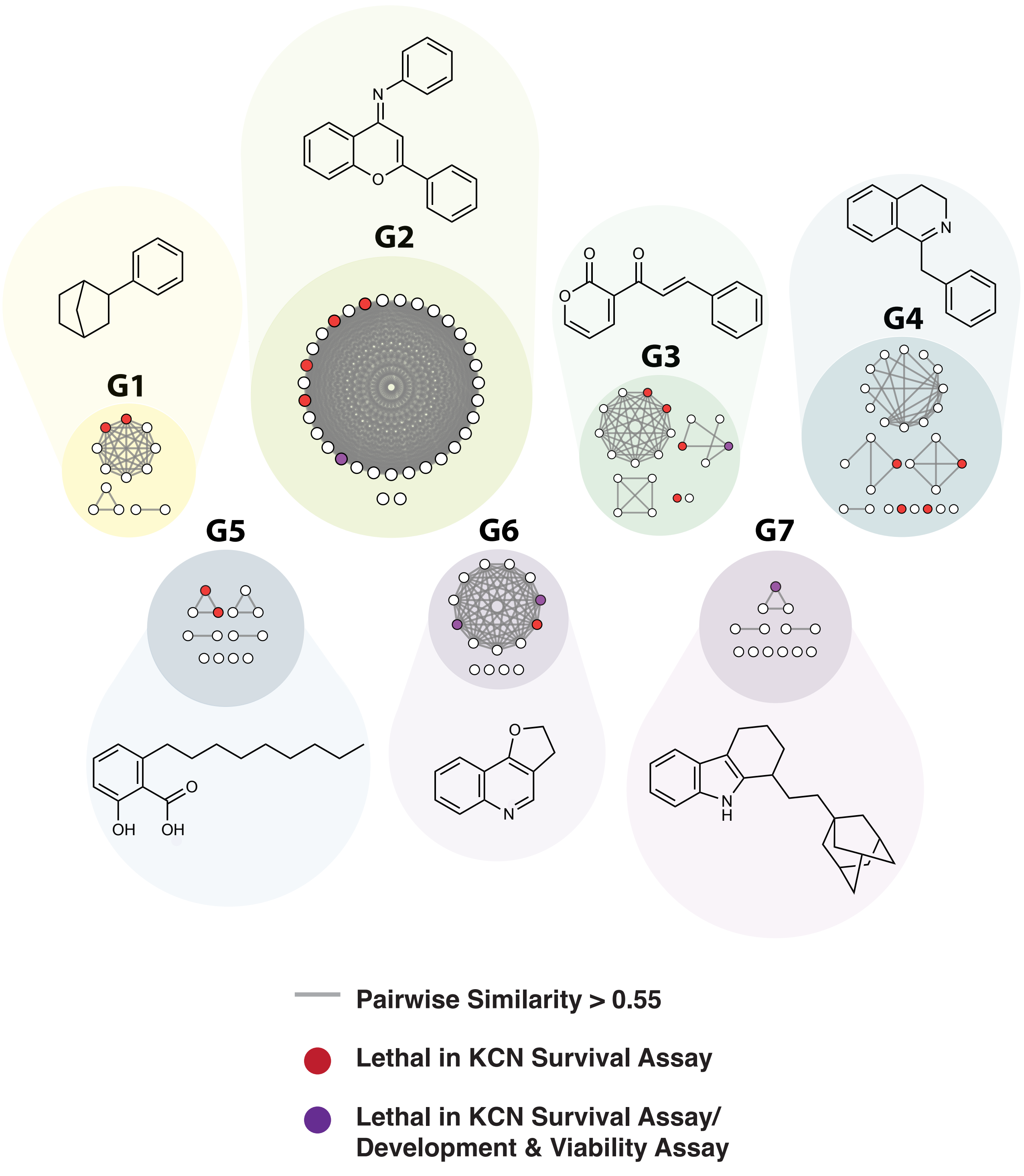

Supplementary Figure 3.

Davie et al.

Supplementary Figure 3. Structural similarity of hits and associated derivatives/analogs from RIKEN NPD libraries. Structural groups corresponding to the seven Pilot library hits in the KCN survival assay screen were determined by RIKEN NPDepo through chemoinformatic clustering of a diverse collection of ~25,000 natural products and derivatives. A network based on chemical similarity of scaffolds was constructed to further classify the 137 compounds within these seven structural groups. Nodes represent molecules, and edges connect molecules within a structural group that have a pairwise Tanimoto coefficient > 0.55. The structural class to which each molecule belongs is indicated by the background colour, while the molecules effect in either assay of *C. elegans* viability is indicated by the node fill colour. Above each class is the Murcko scaffold (for ring systems > 1) of the initial hit identified in the chemical screen.

Supplementary Figure 3 (continued).

RIKEN Structural Groups

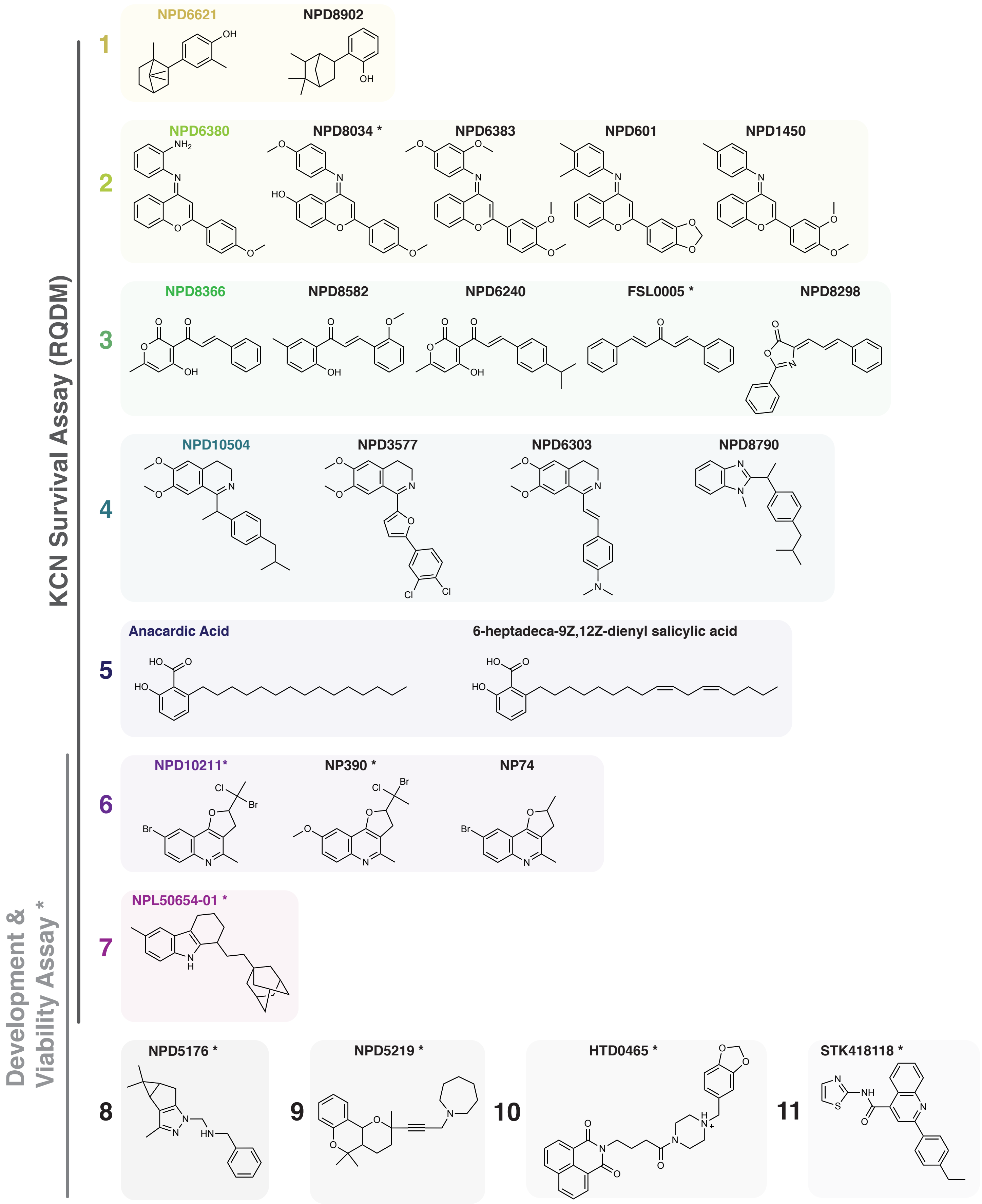

Supplementary Figure 4.

Davie et al.

Supplementary Figure 4. Chemical structures of hits from primary and derivative screens of RIKEN NPD libraries. Chemical structures of all RIKEN NPDepo compounds that were hits in either of the two *C. elegans* viability assays. Chemical structures are arranged and colour-coded based on the structural group to which they belong. The first structure within each structural group represents the initial hit from the Pilot library screen, while additional molecules represent hits from further screens of derivatives, analogs, and other related molecules. Structural groups 1-5 were identified in the KCN survival assay, groups 6-7 were identified in both the KCN survival assay and the development/viability assay, and groups 8-11 were identified in the development and viability assay alone. No analogs and/or derivatives have been tested for groups 8-11, they currently represent singletons. Asterisks denote compounds with activity in the development/viability assay.

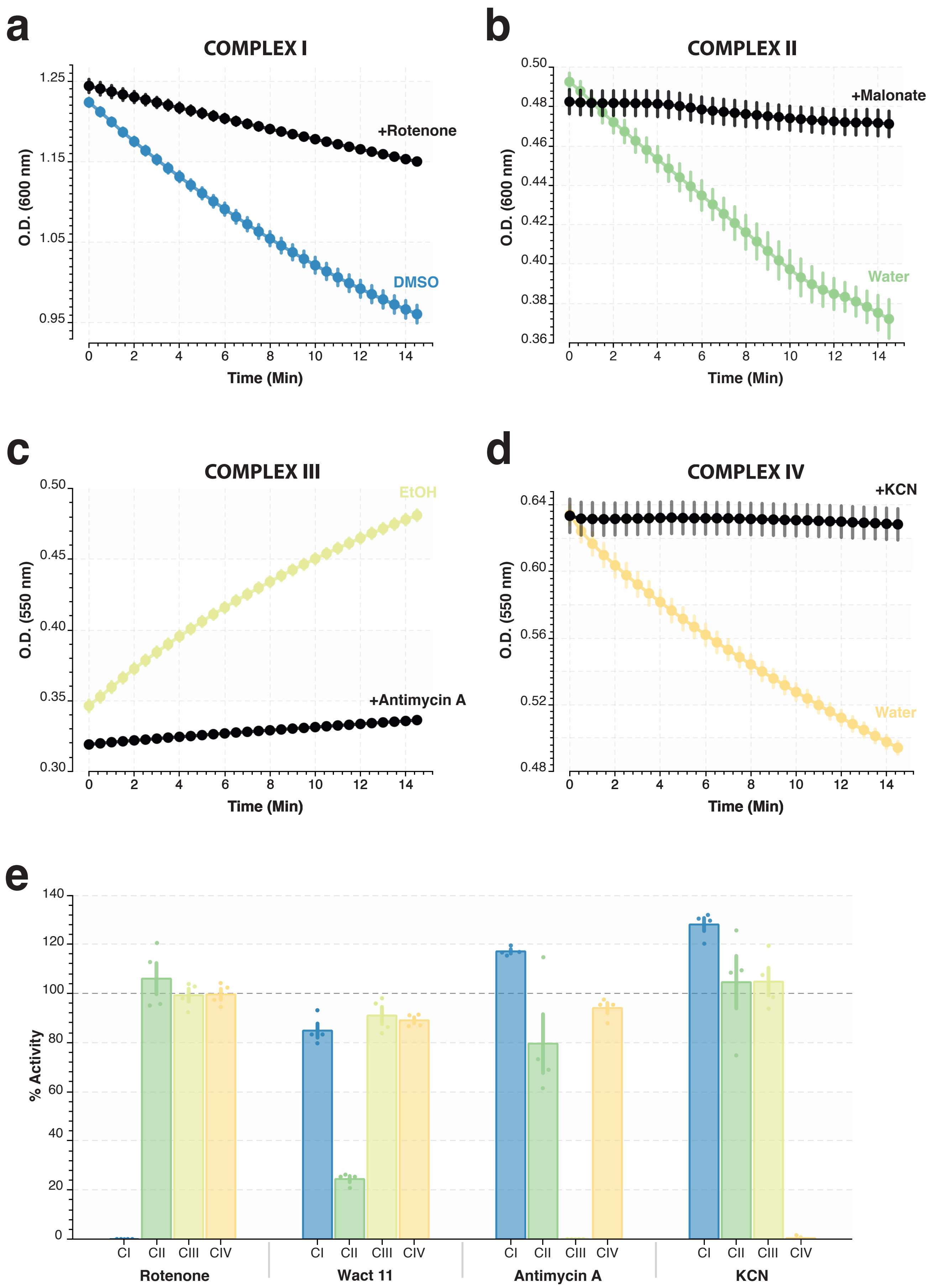

Supplementary Figure 5.

Davie et al.

Supplementary Figure 5. Specific *in vitro* enzymatic activity assays for each of the 4 complexes of the ETC. a-d. Specific enzymatic activity assays performed for each of the 4 complexes of the electron transport chain using mitochondria isolated from adult wild-type *C. elegans* (N2) (see Methods): (**a**) complex I, (**b**) complex II, (**c**) complex III, and (**d**) complex IV. Enzymatic activity was determined spectrophotometrically by measuring the rate of change in absorbance resultant from either the reduction or oxidation of a colorimetric dye (DCIP [600nm], complexes I & II; cytochrome C [550nm], complexes III & IV). Plots show the change in absorbance over time (i.e., activity) in response to solvent or a known inhibitor (black) of each complex (complex I: 10 μM rotenone; complex II: 100 mM malonate; complex III: 10 μM antimycin A; complex IV: 300 μM KCN). **e.** Percent activity of each of the 4 complexes of the electron transport chain in response to pharmacological perturbation with 100 μM of known ETC inhibitors. Percent activity of a complex in response to an inhibitor was calculated relative to the corresponding solvent control. All data are mean of at least three biological replicates; error bars represent SEM.

Supplementary Figure 5 (continued).

Davie et al.

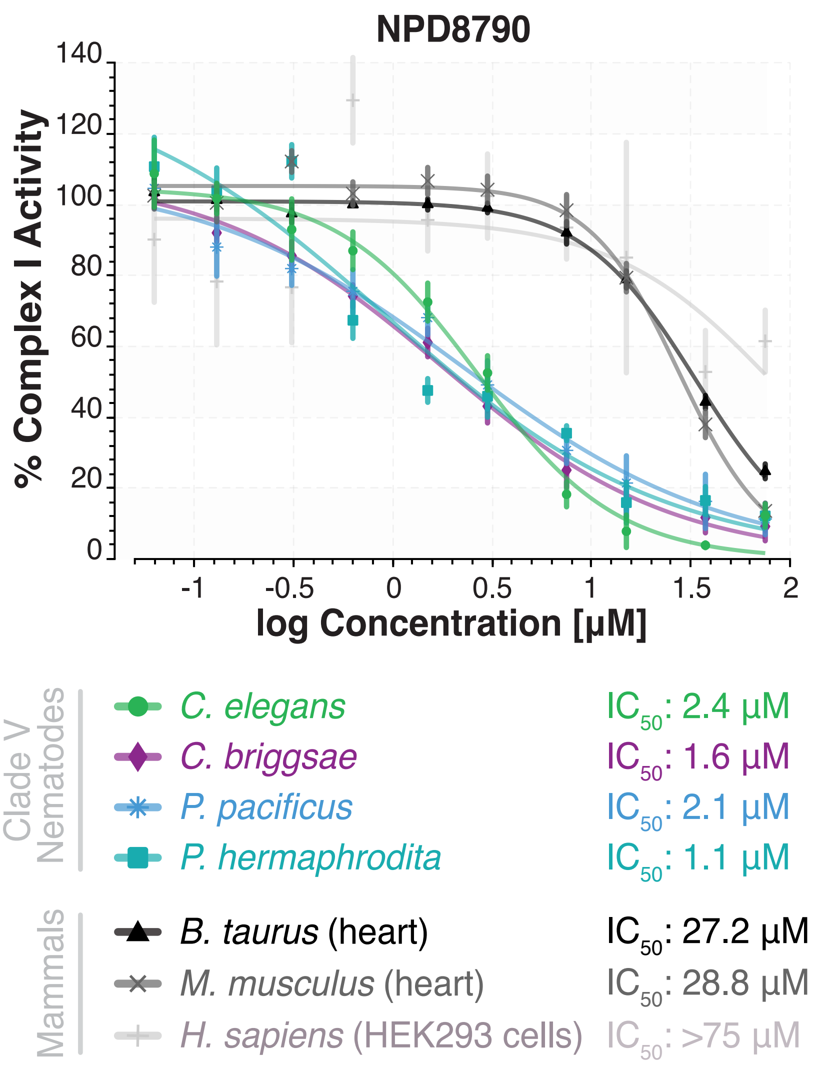

Supplementary Figure 6. NPD8790 demonstrates species-selective inhibition of mitochondrial NADH:quinone oxidoreductase (complex I) *in vitro*. Dose-response curves of NPD8790 against the *in vitro* complex I activity from *C. elegans*, *C. briggsae*, *P. pacificus*, *P. hermaphrodita*, *B. taurus* (heart), *M. musculus* (heart), and *H. sapiens* (HEK293 cells) mitochondria. IC50 values estimated from fitted curves are displayed for each compound. Data are the mean of at least three biological replicates; error bars represent SEM.

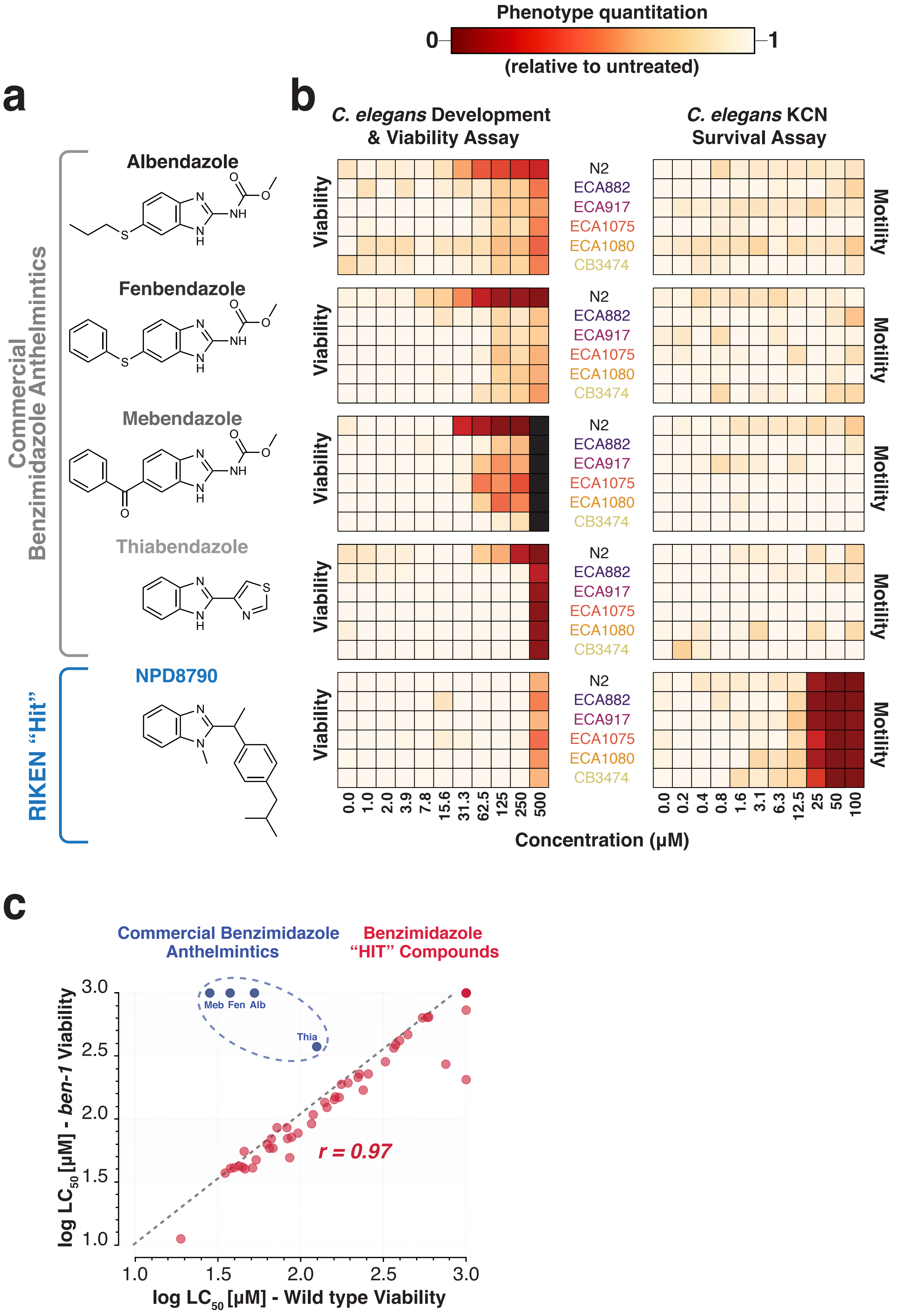

Supplementary Figure 7. Davie et al.

Supplementary Figure 7. NPD8790 shows distinct bioactivity from that of existing benzimidazole anthelmintics. a. Chemical structures of four commercial benzimidazole anthelmintics (e.g., albendazole, fenbendazole, mebendazole, and thiabendazole) and NPD8790. **b.** Dose response of four commercial benzimidazole anthelmintics and NPD8790 in two assays of *C. elegans* viability for six different *C. elegans* strains: N2, wild type; ECA882, deletion (*ben-1*); ECA917, F200Y (*ben-1*); ECA1075, F167Y (*ben-1*); ECA1080, E198A (*ben-1*); CB3474, G104D (*ben-1*). The colour-coded scale denotes the phenotypic outcome of drug treatment (e.g., motility or viability) relative to DMSO controls; white/pale-yellow indicates *C. elegans* growth and viability similar to DMSO controls and red indicates *C. elegans* death and arrested development. **c.** Dose responses for 51 NPD8790 analogs (red) and four commercial benzimidazole anthelmintics (blue) were conducted in *C. elegans* development and viability assays for wild type (N2) and *ben-1* mutant (ECA882) *C. elegans* (see Supplementary Date File 1). LC_{50} values estimated from dose-response curves for each compound on wild-type and *ben-1* mutant worms are displayed as points on the plot. Dotted line displayed has a slope of 1. Pearson's correlation = 0.97 was calculated for the relationship between $\log_{10} LC_{50}$ values of NPD8790 analogs on wild-type and *ben-1* mutant *C. elegans*. All data used for generation of dose-response curves is the mean of at least three biological replicates.

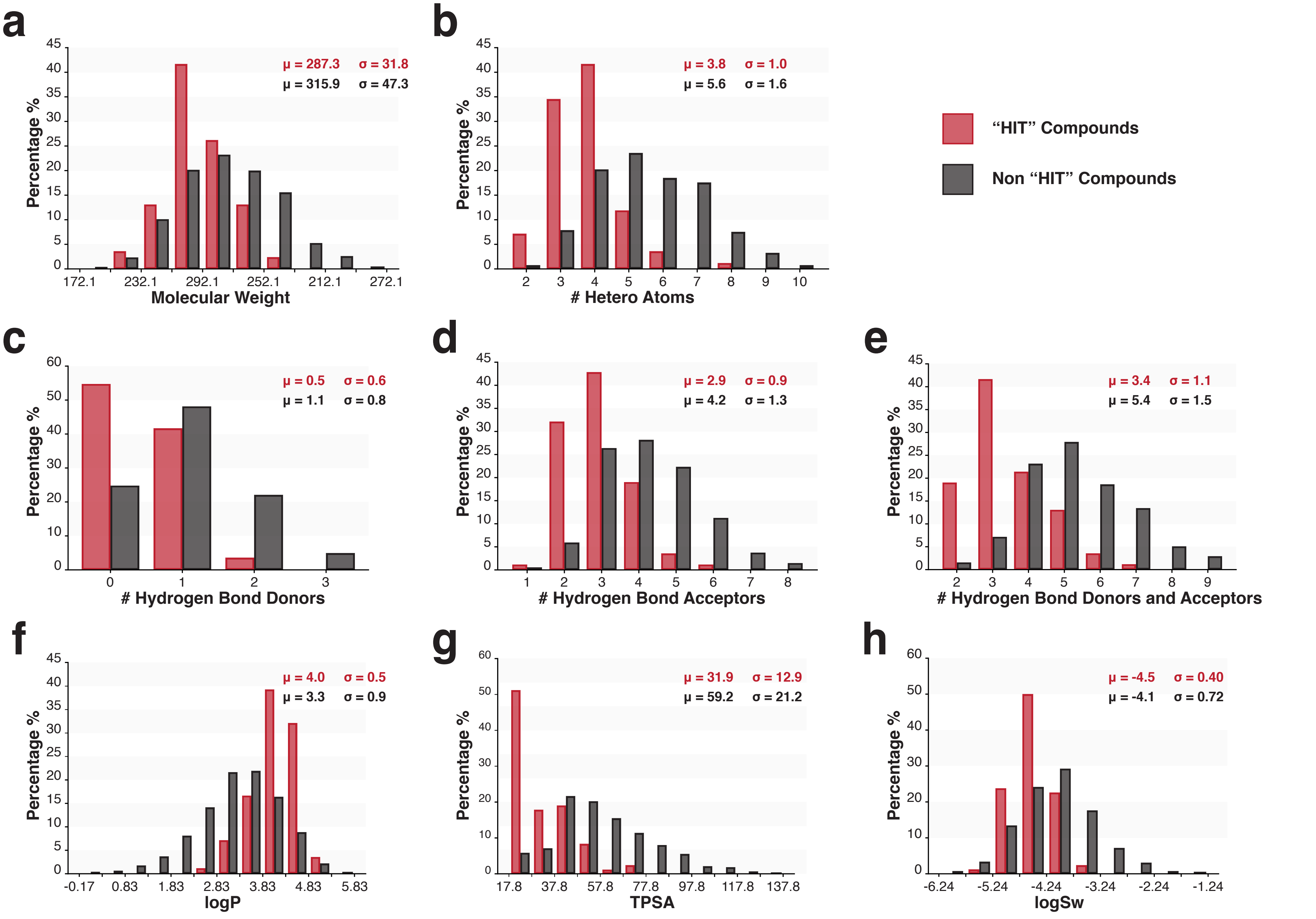

Supplementary Figure 8.

Davie et al.

Supplementary Figure 8. Physicochemical properties of benzimidazole compounds tested in a *C. elegans* assay of RQ-dependent metabolism. Histograms showing distributions of molecular properties for 1,280 benzimidazole compounds that are either active (red) or inactive (black) at 50 μM in the *C. elegans* RQ-dependent KCN survival assay: (**a**) molecular weight, (**b**) number of hetero atoms, (**c**) number of hydrogen bond donors, (**d**) number of hydrogen bond acceptors, (**e**) number of hydrogen bond donors and acceptors, (**f**) logP (hydrophobicity), (**g**) topological polar surface area (TPSA), and (**h**) logSw (solubility). The mean (μ) and standard deviation (σ) for the distributions of both active and inactive benzimidazole compounds are displayed for each molecular property.

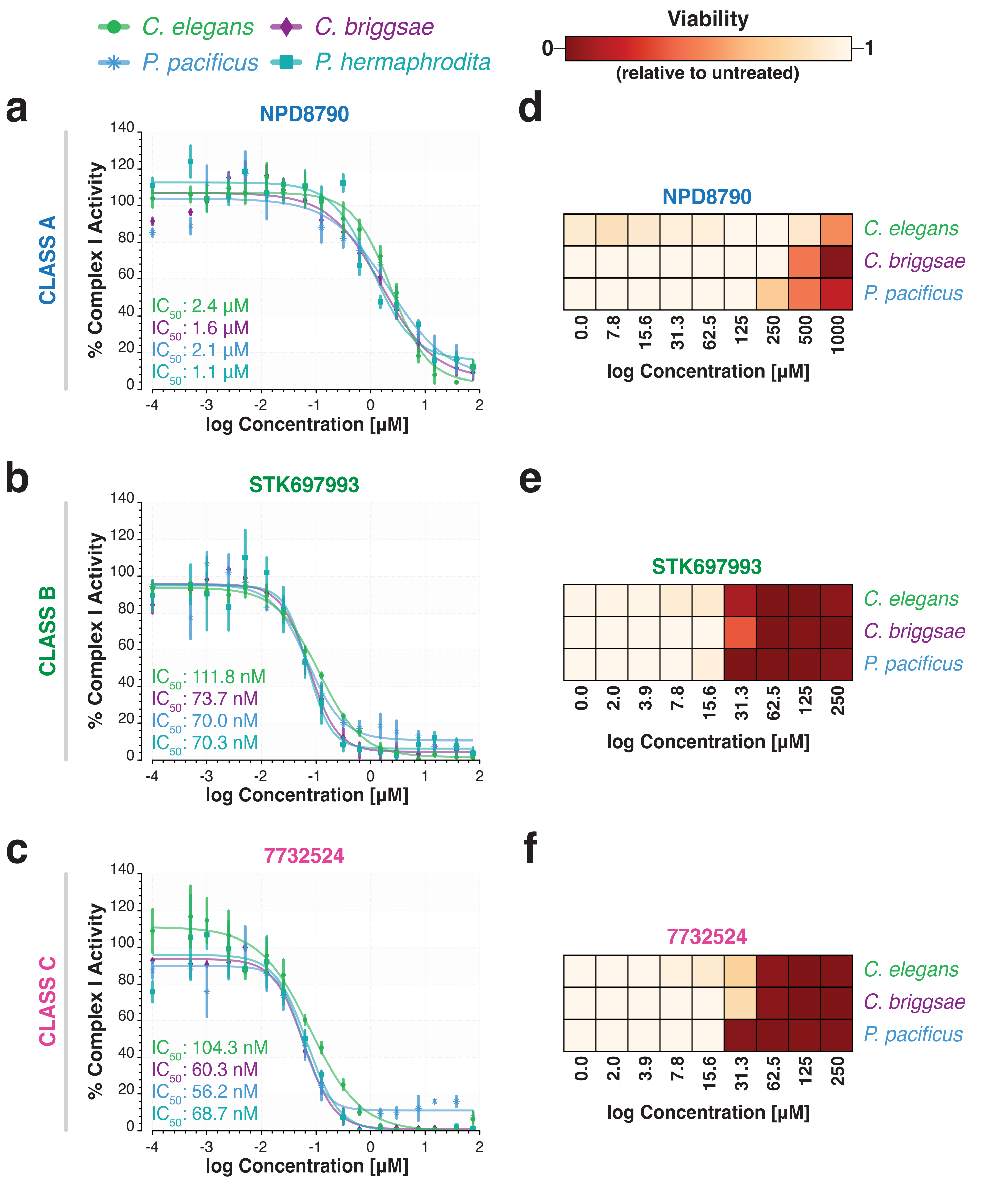

Supplemental Figure 9. **Davie et al.**

Supplementary Figure 9. Class A, B, and C benzimidazoles inhibit complex I of several Clade V nematode species *in vitro*. Dose-response curves of (**a**) NPD8790, (**b**) STK697993, and (**c**) 7732524 against the *in vitro* complex I activity from *C. elegans*, *C. briggsae*, *P. pacificus*, and *P. hermaphrodita* mitochondria. IC_{50} values estimated from fitted curves are displayed for each combination of mitochondria and inhibitor. Data are the mean of at least three biological replicates; error bars represent SEM. Corresponding dose responses for (**d**) NPD8790, (**e**) STK697993, and (**f**) 7732524 against the viability of L1 *C. elegans* (N2), *C. briggsae* (AF16), and *P. pacificus* (PS312) in a nematode liquid growth and development assay. The colour-coded scale denotes the viability of nematodes following drug treatment relative to DMSO controls; white/pale-yellow indicates nematode growth and viability similar to DMSO controls and red indicates nematode death and arrested development. Data are the mean of at least three biological replicates. Due to seemingly poor growth in liquid culture, *P. hermaphrodita* was not tested in the liquid viability and development assay.

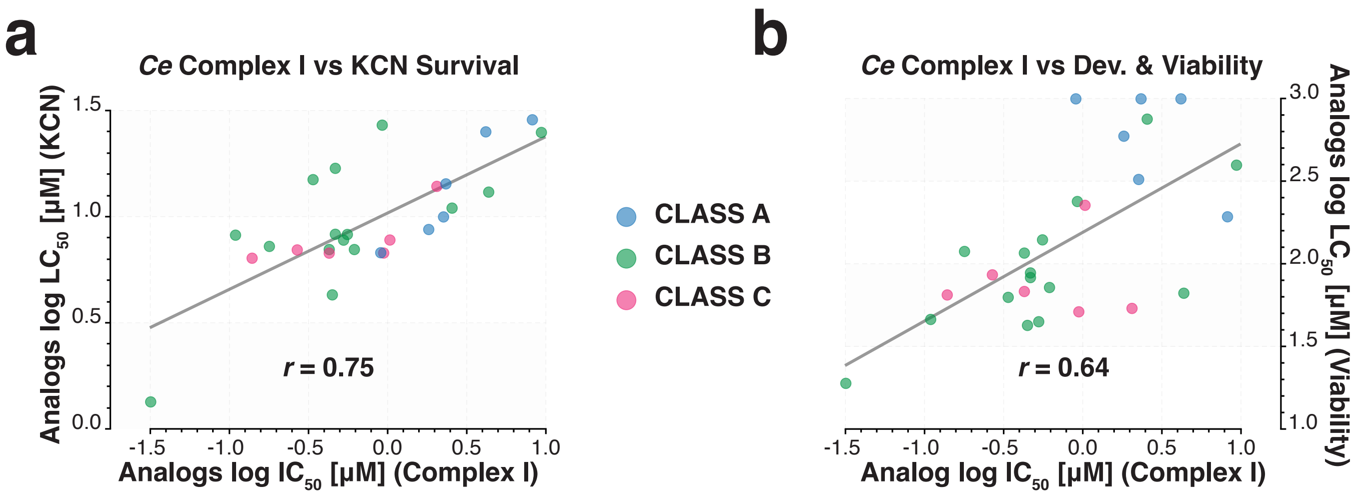

Supplementary Figure 10. Chemical inhibition of complex I activity *in vitro* by Class A, B, and C benzimidazoles is correlated with lethal activity in the *C. elegans* RQ-dependent metabolism assay *in vivo*. Relationship between *in vitro* complex I IC_{50} values and *in vivo C. elegans* LC_{50} values for Class A, B and C benzimidazole analogs in KCN survival assay (**a**) and development/ viability assay (**b**). Pearson's correlation coefficients of 0.75 and 0.64 were determined for the KCN survival assay (**a**) and development/viability assay (**b**), respectively. All IC_{50} and LC_{50} values used for analyses were estimated from fitted dose-response curves (see Supplementary Data File 1). All data used for generation of dose-response curves are the mean of at least two biological replicates.

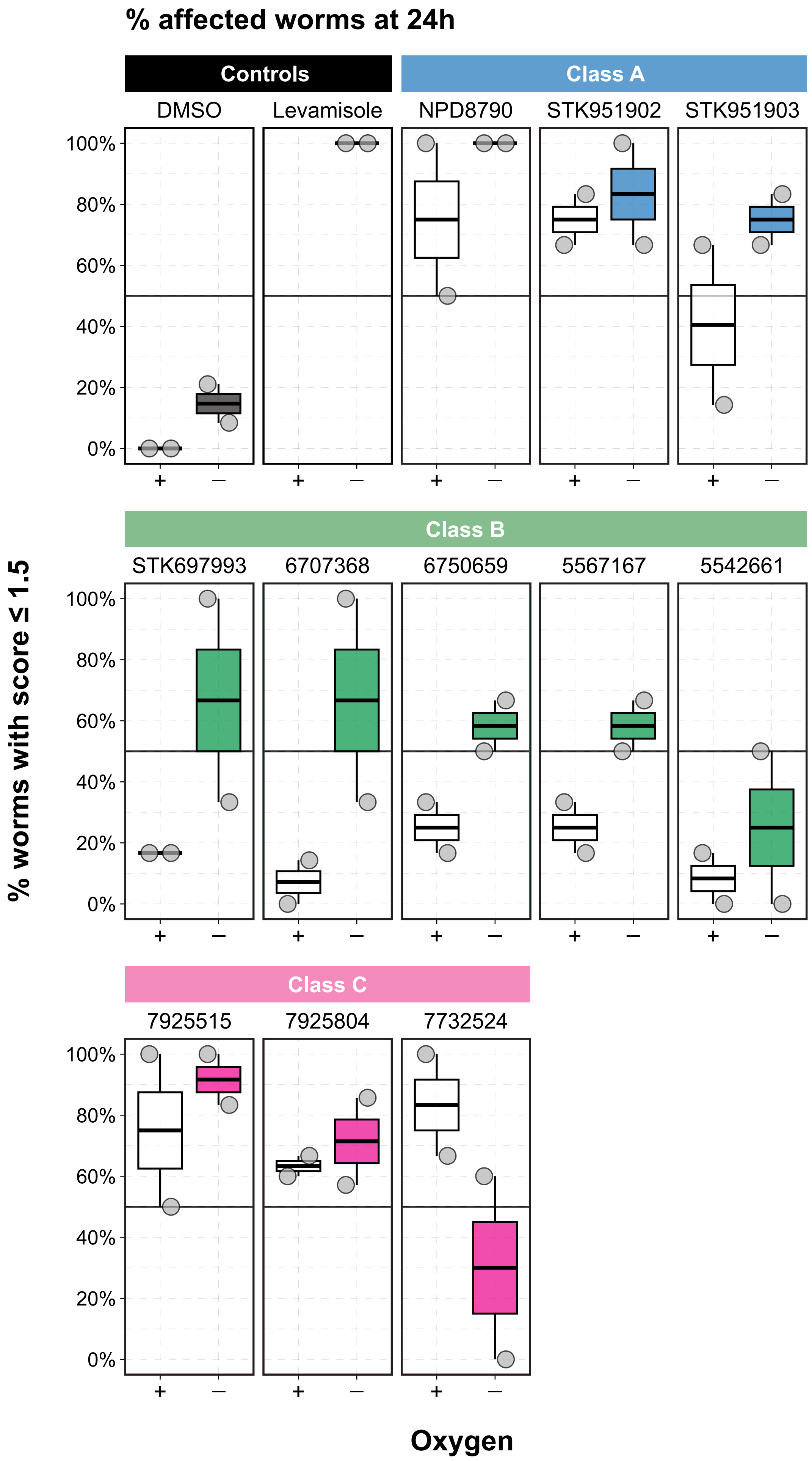

Supplementary Figure 11. **Davie et al.**

Supplementary Figure 11. Anaerobic environments increase the effects of benzimidazole complex I inhibitors against adult *H. polygyrus* viability *in vitro*. The activities of eleven benzimidazole derivatives were tested against adult *H. polygyrus* viability *in vitro* under aerobic (+, white boxplots) and anaerobic (–, coloured boxplots) conditions (see Methods). After 24-hour chemical treatment, worm viability was manually scored by an examiner using a scale from 0 (dead) to 3 (motile); DMSO was used as a negative control in both aerobic and anaerobic conditions; levamisole was used as a positive control in anaerobic conditions. The percentage of affected worms (i.e., worms with a score equal to or lower than 1.5) in each biological replicate is shown; data are from two biological replicates. Within each boxplot, black horizontal lines denote group medians, boxes span from the 25th to the 75th percentile of each group's distribution, and whiskers extend to the maximum and minimum values.

Supplementary Table 1. Selectivity of *C. elegans* complex I inhibition. NPD8790 dose response data (IC_{50} values) against *in vitro* complex I activity from wild-type *C. elegans* (N2), *M. musculus* heart and *B. taurus* heart mitochondria. Dose response data for 10 additional compounds with complex I inhibitory activity are included for comparison. IC_{50} values were estimated from fitted dose-response curves; all dose response data are the mean of at least three biological replicates. "-" indicate values that were not determined.

Compound ID	*C. elegans* complex I IC_{50} (μM)	*M. musculus* complex I IC_{50} (μM)	*B. taurus* complex I IC_{50} (μM)	*M. musculus: C. elegans* IC_{50} Fold Change	*B. taurus: C. elegans* IC_{50} Fold Change
NPD8790	2.35	28.86	27.23	12.3	11.6
NPD10504	3.29	9.52	11.26	2.9	3.4
NPD3577	12.08	18.77	9.49	1.6	0.8
NPD6303	11.91	34.92	34.29	2.9	2.8
NPD390	35.21	53.55	-	1.5	-
NPL50654-01	13.32	13.04	-	1.0	-
NPD8902	67.14	>75	-	1.1	-
Rotenone	0.045	0.037	0.046	0.8	1.0
Papaverine	4.34	4.58	8.50	1.1	1.9
Fenazaquin	0.0042	0.021	0.024	5.0	5.7
Fenpyroximate	0.021	-	0.048	-	2.3

Supplementary Table 2. Sensitivity of anthelmintic/nematicide-resistant strains to Class A, B, and C benzimidazole complex I inhibitors. Dose response data (LC_{50}) for NPD8790 (Class A), STK697993 (Class B), and 7732524 (Class C) against L1 wild-type *C. elegans* (N2) and 7 anthelmintic/nematicide-resistant mutant strains. Data for rotenone, the classical complex I inhibitor is included for comparison. LC_{50s} were estimated from fitted dose-response curves; all dose response data are the mean of at least three biological replicates.

Genotype	Resistance	*C. elegans* Development & Viability LC_{50} (μM)			
		Rotenone	NPD8790 (Class A)	STK697993 (Class B)	7732524 (Class C)
Wild type	None	3.1	>1000	31.5	48.4
ben-1(e1880)	Benzimidazoles (e.g., albendazole)	3.4	>1000	38.6	50.0
unc-29(e193)	Nicotinic acetylcholine receptor agonists (e.g., levamisole, pyrantel, tribendimidine)	1.5	>1000	22.9	37.8
bre-1(ye4)	*B. thuringiensis* crystal (Cry) proteins	3.5	>1000	31.1	41.2
acr-23(ok2804)	Amino-acetonitrile derivatives (AADs; e.g., monepantel)	2.1	>1000	34.9	50.2
avr-14(ad1302); avr-15(ad1051); glc-1(pk54)	Macrocyclic lactones (e.g., ivermectin, moxidectin)	1.4	>1000	28.3	46.5
slo-1(js379)	Cyclodepsipeptides (e.g., emodepside)	1.0	176.7	16.1	31.4
mev-1(tr355)	Fluopyram	3.2	>1000	28.5	42.9

Supplementary Table 3. Summary of small molecule screening data.

Category	Parameter	Description
Assay	Type of assay	*C. elegans* (whole organism) chemical screen for molecules with anthelmintic potential
	Target	Rhodoquinone-dependent (anaerobic) metabolism of nematodes
	Primary measurement	*C. elegans* motility through automated imaging
	Key reagents	200 µM potassium cyanide (KCN)
	Assay protocol	*C. elegans* L1 larvae are incubated with a combination of small molecule and 200 µM KCN for 15 hours. After 15 hours, KCN is diluted with M9 buffer and worms are allowed to recover from KCN treatment for 3 hours. After 3 hours, worm survival and overall health is assessed relative to untreated controls through measurement of worm motility
	Additional comments	
Library	Library size	480
	Library composition	Authentic library (80 natural products and derivatives with previously characterized activity); Pilot library (400 uncharacterized natural products and derivatives, each representative of a structurally related family of compounds from a much larger and diverse set of ~25,000 natural product derivatives)
	Source	RIKEN Natural Product Depository (NPDepo)
	Additional comments	
Screen	Format	96-well microplates
	Concentration(s) tested	50 µM (in DMSO)
	Plate controls	16 DMSO controls per plate (Columns 1 & 12)
	Reagent/ compound dispensing system	Multi-channel pipette and/or pinning tool
	Detection instrument and software	Nikon Ti Eclipse inverted microscope (using Nikon NIS Elements AR software)
	Assay validation/QC	Known bioactive compounds from the Authentic library were used to validate the assay
	Correction factors	N/A
	Normalization	Raw motility scores for each chemical plate were normalized to motility of DMSO controls on each plate. Modified Z-scores were calculated from normalized motility scores based on a plate-to-plate basis using the median and median absolute deviation (MAD)
	Additional comments	Data from screen are provided in the Source Data File
Post-HTS analysis	Hit criteria	Modified Z-scores < -3 i.e., deviating more than 3X the MAD from the median of each plates
	Hit rate	~2%
	Additional assay(s)	Hit compounds were reordered for dose responses in original assay and counter screened in a HEK293 cell viability assay
	Confirmation of hit purity and structure	Hit compounds are commercially available and were reordered from Vitas-M chemical supplier for retesting
	Additional comments	Data from screen are provided in the Source Data File
