# Supplementary material for: Identification of a family of species-selective complex I inhibitors as potential anthelmintics
Source: Nat Commun. 2024 May 8;15:3367. doi: 10.1038/s41467-024-47331-3 (PMC11079024; doi:10.1038/s41467-024-47331-3)
Supplement: Supplementary file 2 — Reporting Summary [file 41467_2024_47331_MOESM2_ESM.pdf]

# nature portfolio

Corresponding author(s): Andrew Gordon Fraser

Last updated by author(s): Mar 4, 2024

# Reporting Summary

Nature Portfolio wishes to improve the reproducibility of the work that we publish. This form provides structure for consistency and transparency in reporting. For further information on Nature Portfolio policies, see our Editorial Policies and the Editorial Policy Checklist.

## Statistics

For all statistical analyses, confirm that the following items are present in the figure legend, table legend, main text, or Methods section.

| n/a | Confirmed | |
|---|---|---|
| ☐ | ☒ | The exact sample size (*n*) for each experimental group/condition, given as a discrete number and unit of measurement |
| ☐ | ☒ | A statement on whether measurements were taken from distinct samples or whether the same sample was measured repeatedly |
| ☐ | ☒ | The statistical test(s) used AND whether they are one- or two-sided<br>*Only common tests should be described solely by name; describe more complex techniques in the Methods section.* |
| ☒ | ☐ | A description of all covariates tested |
| ☒ | ☐ | A description of any assumptions or corrections, such as tests of normality and adjustment for multiple comparisons |
| ☐ | ☒ | A full description of the statistical parameters including central tendency (e.g. means) or other basic estimates (e.g. regression coefficient) AND variation (e.g. standard deviation) or associated estimates of uncertainty (e.g. confidence intervals) |
| ☒ | ☐ | For null hypothesis testing, the test statistic (e.g. *F*, *t*, *r*) with confidence intervals, effect sizes, degrees of freedom and *P* value noted<br>*Give P values as exact values whenever suitable.* |
| ☒ | ☐ | For Bayesian analysis, information on the choice of priors and Markov chain Monte Carlo settings |
| ☒ | ☐ | For hierarchical and complex designs, identification of the appropriate level for tests and full reporting of outcomes |
| ☐ | ☒ | Estimates of effect sizes (e.g. Cohen's *d*, Pearson's *r*), indicating how they were calculated |

*Our web collection on statistics for biologists contains articles on many of the points above.*

## Software and code

Policy information about availability of computer code

| | |
|---|---|
| Data collection | C. elegans In vivo KCN survival and viability data \| Nikon NIS Elements AR (v. 3.22.11)<br>HEK293 cell data \| BMG Labtech CLARIOStar (v. 5.40 R2)<br>ETC enzymatic activity data \| Thermofisher VarioSkan SkanIt (v. 6.0.2)<br>All other data were collected manually. |
| Data analysis | C. elegans In vivo KCN survival and viability data \| Quantification of C. elegans images were calculated using previously published custom python scripts (https://doi.org/10.1534/g3.118.200374)<br>HEK293 cell data & ETC enzymatic activity data \| Raw absorbance data from plate readers were exported to Microsoft Excel for Mac (v. 16.66.1)<br>Organization and analysis of all raw data was conducted using Python (v. 3.7.4) using the NumPy (v. 1.17.2) and SciPy (v. 1.3.1) libraries, dose-response curves were determined using the Python rpy2 interface (v. 2.9.4) and the R (v. 3.6.1) "drc" package (v. 2.9.4), visualization of time-course and dose-response data (e.g. line graphs, dose-response curves, heatmaps) was performed using the Matplotlib (v. 3.1.3), Seaborn (v. 0.12.2), and Bokeh (v. 1.3.4) Python libraries, and adobe illustrator (v. 27.8)<br>Chemoinformatics - Chemical structures and similarity \| Murcko scaffolds of chemicals were calculated in DataWarrior (v. 5.5.0), scaffold FP2 fingerprints and measurements of chemical similarity were determined in Python (v. 3.7.4) using the Pybel library (v. 3.0.1), visualization of chemical similarity network was conducted in Cytoscape (v. 3.8.0) and adobe illustrator (v. 27.8) |

For manuscripts utilizing custom algorithms or software that are central to the research but not yet described in published literature, software must be made available to editors and reviewers. We strongly encourage code deposition in a community repository (e.g. GitHub). See the Nature Portfolio guidelines for submitting code & software for further information.

## Data

Policy information about availability of data

All manuscripts must include a data availability statement. This statement should provide the following information, where applicable:

- Accession codes, unique identifiers, or web links for publicly available datasets
- A description of any restrictions on data availability
- For clinical datasets or third party data, please ensure that the statement adheres to our policy

Data Availability
A data availability statement is included in the manuscript. Raw data are provided in the Source Data file for Figs. 1-7, Tables 1-3, and all Supplementary Figs (1-11), Tables (1 & 2), and Files (1 &2). Any other material or data that support the conclusions of this manuscript are available upon request from the corresponding author AGF. There are no restrictions on data availability.

## Research involving human participants, their data, or biological material

Policy information about studies with human participants or human data. See also policy information about sex, gender (identity/presentation), and sexual orientation and race, ethnicity and racism.

| | |
|---|---|
| Reporting on sex and gender | This study did not involve human participants. |
| Reporting on race, ethnicity, or other socially relevant groupings | This study did not involve human participants. |
| Population characteristics | This study did not involve human participants. |
| Recruitment | This study did not involve human participants. |
| Ethics oversight | This study did not involve human participants. |

Note that full information on the approval of the study protocol must also be provided in the manuscript.

# Field-specific reporting

Please select the one below that is the best fit for your research. If you are not sure, read the appropriate sections before making your selection.

☒ Life sciences ☐ Behavioural & social sciences ☐ Ecological, evolutionary & environmental sciences

For a reference copy of the document with all sections, see nature.com/documents/nr-reporting-summary-flat.pdf

# Life sciences study design

All studies must disclose on these points even when the disclosure is negative.

| | |
|---|---|
| Sample size | No statistical methods were used to predetermine sample size. Throughout all experiments, at least two biological replicates each with two or more technical replicates were performed for all in vitro assays and at least three biological replicates were performed for all free-living nematode assays. The number of replicates for each experiment is indicated in the Material and Methods section as well as the corresponding figure legends. Sample sizes were selected to demonstrate reproducibility of results and permit calculation of experimental error, while also balancing the feasibility of data collection/analysis (cost, time, etc.) and ethical considerations (mice used). As can be observed in figures, the overall variation between experiments was relatively low and the results were highly reproducible between replicates. Additionally, the primary focus of this paper was on results with large effect sizes, which was able to be captured with the smaller samples sizes that are feasible in biological research. We feel this demonstrates that the sample sizes chosen for each assay was sufficient. |
| Data exclusions | No data were excluded from this study. |
| Replication | At least two biological replicates each with two or more technical replicates were performed for all experiments; in many cases more replicates were performed. For both in vitro and in vivo experiments, variation between replicates was very low. All experimental measurements were reproducible and corresponding statistical analyses were performed as described in the Materials and Methods section. In order to minimize variation between experimental replicates, covariates such as temperature, humidity, age, developmental stage, media/buffer composition, and examiners were held constant. |
| Randomization | In all experiments, test subjects (organisms, cells, etc.) were randomly assigned and distributed amongst test conditions. There was no attempt to bias samples by subjectively picking or assigning specific test subjects to particular conditions. |
| Blinding | For all experiments, examiners were not blinded during data collection and/or analysis. For C. elegans, HEK293 cell, and mitochondrial assays, blinding was not necessary as experiments were quantified by applying a uniform set of automated techniques/protocols on collected images and/or absorbance measurements (not influenced by examiner). For in vitro and in vivo testing of H. polygyrus, blinding was not performed because only one person was available for experimentation. |

# Reporting for specific materials, systems and methods

We require information from authors about some types of materials, experimental systems and methods used in many studies. Here, indicate whether each material, system or method listed is relevant to your study. If you are not sure if a list item applies to your research, read the appropriate section before selecting a response.

## Materials & experimental systems

| n/a | Involved in the study | |
|---|---|---|
| x | | Antibodies |
| | x | Eukaryotic cell lines |
| x | | Palaeontology and archaeology |
| | x | Animals and other organisms |
| x | | Clinical data |
| | | Dual use research of concern |
| x | | Plants |

## Methods

| n/a | Involved in the study | |
|---|---|---|
| x | | ChIP-seq |
| x | | Flow cytometry |
| x | | MRI-based neuroimaging |

# Eukaryotic cell lines

Policy information about cell lines and Sex and Gender in Research

| | |
|---|---|
| Cell line source(s) | HEK293 cells were purchased from Thermofisher (Cat# R71007). |
| Authentication | HEK293 cells were authenticated by STR analysis as the Toronto Hospital for Sick Children authentication facility. |
| Mycoplasma contamination | HEK293 cells were tested regularly for mycoplasma contamination. All tests were performed a short time prior to experimentation to confirm the cell line was negative for mycoplasma contamination. |
| Commonly misidentified lines (See ICLAC register) | The HEK293 cell line is not on the list of commonly misidentified cell lines. |

# Animals and other research organisms

Policy information about studies involving animals; ARRIVE guidelines recommended for reporting animal research, and Sex and Gender in Research

Laboratory animals

Free-living nematode species were used for the bulk of the experimentation, including chemical screening, dose response experiments, as well as the isolation of mitochondria for in vitro enzymatic testing. Experiments were primarily conducted on the model nematode, Caenorhabditis elegans (C. elegans). In addition to the C. elegans wild type strain (N2), this research also featured the use of a variety of  C. elegans mutants strains including: CB1003 (kynu-1(e1003) X); anthelmintic-resistant strains – CB3474 (ben-1(e1880) III), CB193 (unc-29(e193) I), HY496 (bre-1(ye4) IV), RB2119 (acr-23(ok2804) V), JD608 (avr-14(ad1302) I; avr-15 (ad1051) V; glc-1 (pk54) V), NM1968 (slo-1(js379) V), RP2635 (mev-1(tr355) III); benzimidazole-resistant CRISPR strains – ECA882 (ben-1(ean64) III), ECA917 (ben-1(ean98) III), ECA1075 (ben-1(ean143) III), ECA1080 (ben-1(ean148) III). In addition to C. elegans, several closely related clade V nematodes were also used for experimentation including the C. briggsae strain AF16, P. pacificus strain PS312, and P. hermaphrodita strain B178. Experiments on all nematode species and strains were primarily conducted on first larval stage (L1) worms, with the exception that mitochondria were isolated from adult nematodes. All strains were cultured and propagated under standard laboratory conditions , grown either on NGM agar plates seeded with OP50 or in liquid S-media or NGM media with concentrated HB101.

Laboratory mice were used for the isolation of mammalian mitochondria as well as the culture and propagation of the parasitic nematode, Heligmosomoides polygyrus (H. polygyrus).
For isolation of mitochondria, six 8-10 week-old C57Bl/6 female mice (bred and maintained within an animal care facility at the University of Toronto, Canada) were freshly dissected and the hearts were removed and collected.
For in vitro and in vivo studies of H. polygyrus, the life cycle of H. polygyrus worms were maintained in female NMRI mice (bred and maintained at the Swiss Tropical and Public Health Institute, Switzerland). The mice were kept in individually ventilated cages (IVC) under environmentally controlled conditions (temperature: 22°C ± 2°C; relative humidity: 55% ± 15%; artificial lighting with a circadian cycle of 12h of light) and had free access to water (municipal tap water supply) and rodent food. Female three-week old NMRI mice (Charles River, Germany) were allowed to acclimatize to their new environment for one week before infection. Upon two days of arrival, dexamethasone (0.25 mg/L) was added to the normal drinking water for immunosupression to enable parasite establishment. After one week, mice were orally infected with 100 H. polygyrus L3 larvae. For the in vitro larval assay, mice feces were collected two weeks post-infection and eggs were isolated. The eggs were left on agar at room temperature in the dark for eight to ten days to allow them to develop into L3 for testing. For the adult in vitro drug screening assay, female NMRI mice (5 week-old mice; 2 weeks post-infection) were euthanized with CO2 and adult H. polygyrus were dissected out of the intestines for experiments.

Wild animals

The study did not involve any wild animals.

| | |
|---|---|
| Reporting on sex | Maintenance of H. polygyrus and in vivo anthelminthic testing was performed only in female mice for pragmatic reasons - there is significant conflict between male mice when caged that can influence the interpretation the anthelmintic effects of soil-transmitted helminth infections. Based on past assays and experience, there is a small impact of sex on anthelmintic activity, but the effect is very minor. Thus female mice were used to carry out all assays. |
| Field-collected samples | This study did not involve any field-collected samples. |
| Ethics oversight | All animal experimental procedures and husbandry in this study were performed by trained personnel in accordance with either the ethical committee of the canton Basel-Stadt (permission no. 520) and the University of Basel, or the Canadian Council on Animal Care with approval from the Animal Care Committee at the University of Toronto. |

Note that full information on the approval of the study protocol must also be provided in the manuscript.
