# Supplementary material for: Identification of a family of species-selective complex I inhibitors as potential anthelmintics
Source: Nat Commun. 2024 May 8;15:3367. doi: 10.1038/s41467-024-47331-3 (PMC11079024; doi:10.1038/s41467-024-47331-3)
Supplement: Supplementary file 4 — Description of Additional Supplementary Files [file 41467_2024_47331_MOESM4_ESM.pdf]

Supplementary Data File 1. Summary of NPD8790 analog bioactivity. Chemical structures, molecular properties, and a summary of bioactivity for 51/84 NPD8790 analogs identified in benzimidazole analog screen. Dose response (LC_{50}) data is provided for *C. elegans* KCN survival assays, *C. elegans* wild-type and *ben-1* development and viability assays, and HEK293 viability assays. Dose response (IC_{50}) data is also provided for *in vitro* complex I activity assays from wild-type *C. elegans* and *B. taurus* heart mitochondria. IC_{50} and LC_{50} values were estimated from fitted dose-response curves; all dose response data are the mean of at least two biological replicates. "-" indicate values that were not determined.

Supplementary Data File 2. Class A, B, and C benzimidazole structure-activity relationships. Chemical structures and summary of bioactivity for 27 NPD8790 analogs belonging to class A, B, and C benzimidazoles. For each compound, the core structure, and the positions of R_1, R_2, R_3, and R_4 groups are indicated. Dose response (LC_{50}) data is provided for *C. elegans* KCN survival assays, *C. elegans* wild-type and *ben-1* development and viability assays, and HEK293 viability assays. Dose response (IC_{50}) data is provided for *in vitro* complex I activity assays from wild-type *C. elegans* and *B. taurus* heart mitochondria. IC_{50} and LC_{50} values were estimated from fitted dose-response curves; all dose response data are the mean of at least two biological replicates. “-“ indicate values that were not determined.
